# Detecting immunoglobulin G4-related intracranial arteriopathy with magnetic resonance vessel wall imaging: a preliminary experience in two cases

**DOI:** 10.1186/s12883-022-03010-8

**Published:** 2022-12-12

**Authors:** Koki Mitani, Takeshi Funaki, Masahiro Tanji, Hideo Onizawa, Hajime Yoshifuji, Yasutaka Fushimi, Shinya Torimaki, Kazumichi Yoshida, Susumu Miyamoto

**Affiliations:** 1 Department of Neurosurgery, Kyoto University Graduate School of Medicine, 54 Kawahara-cho, Shogoin, Sakyo-ku, 606-8507 Kyoto, Japan; 2 Department of Advanced Medicine for Rheumatic Diseases, Graduate School of Medicine, Kyoto University, Kyoto, Japan; 3 Department of Rheumatology and Clinical Immunology, Kyoto University Graduate School of Medicine, Kyoto, Japan; 4 Department of Diagnostic Imaging and Nuclear Medicine, Kyoto University Graduate School of Medicine, Kyoto, Japan

**Keywords:** CNS vasculitis, IgG4-related disease, Magnetic resonance imaging, Vessel wall imaging, DANTE, Case report

## Abstract

**Background:**

Detecting immunoglobulin G4 (IgG4)-related intracranial arteriopathy, a rare neurovascular complication of IgG4-related disease, is challenging. While magnetic resonance (MR) vessel wall imaging (VWI) can visualize various neurovascular pathologies, its application to this arteriopathy has not been reported as of this writing.

**Case presentation:**

A 74-year-old male and a 65-year-old female manifested multiple cranial nerve palsy and neck pain, respectively. Both cases exhibited multiorgan masses with markedly elevated serum IgG4 levels and were clinically diagnosed with IgG4-related disease. Three-dimensional T1-weighted black blood VWI with and without contrast agent identified intracranial vascular lesions characterized as nearly-circumferential mural thickening with homogeneous contrast enhancement in the internal carotid and vertebral arteries; some of the lesions had been unrecognized with screening MR angiography due to expansive remodeling. The former patient underwent corticosteroid therapy, and VWI after treatment revealed decreased mural thickening and enhancement.

**Conclusion:**

Further studies to elucidate characteristic findings of VWI might contribute to early detection of this treatable pathology.

## Background

Immunoglobin G4-related disease (IgG4-RD) is a multiorgan immune-mediated condition [1]. It is characterized by an elevated concentration of serum IgG and dense infiltration of IgG4-positive plasmacyte accompanied by fibrosis [2]. It commonly causes autoimmune pancreatitis, Mikulicz’s disease (dacryoadenitis with enlargement of the parotid and submandibular glands), and tubulointerstitial nephritis [1, 3]. Infundibular hypophysitis, hypertrophic pachymeningitis, and cranial nerve palsy associated with intracerebral inflammatory pseudotumor are well-known intracranial pathologies [4, 5].

While the aorta and coronary artery are the common vascular sites involved in this disease [6], only a few reports have described intracranial vascular lesions [7–11]. The optimal imaging modality for detecting such lesions remains unclear due to its rare occurrence. Early detection of such lesions is essential because this disease is treatable with corticosteroid [1, 12]. Here, we describe two cases with intracranial arterial lesions associated with IgG4-RD. They exhibited characteristic findings on magnetic resonance (MR) vessel wall imaging (VWI) with a recently developed preparation module, the delay alternating with nutation for tailored excitation (DANTE) prepulse [13–15].

## Case presentation

### Case 1

#### History

**Fig. 1:**
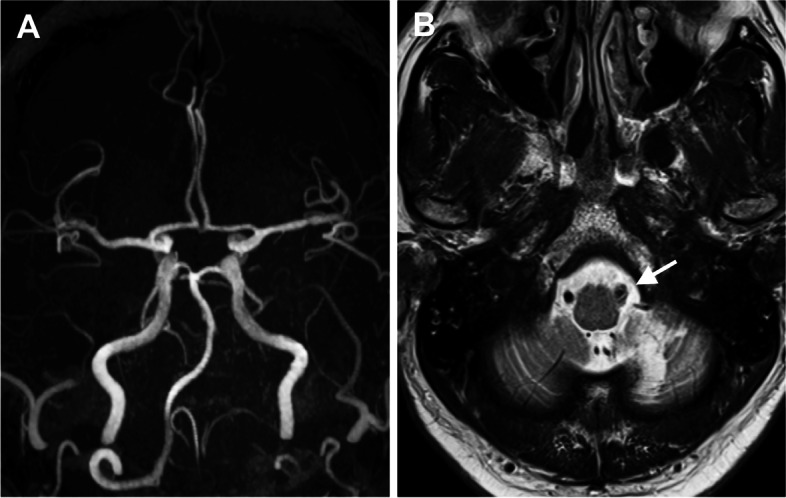
Magnetic resonance (MR) images on admission (Case 1). **A** Time-of-flight MR angiography revealing occlusion of the left vertebral artery. **B** T2-weighted image mimicking left vertebral dissection (arrow)

A 74-year-old male, who complained of left facial palsy, diplopia, and dysphagia, was referred to our department. The neurological examination revealed peripheral facial palsy of House-Brackman grade III and positive curtain sign at the left soft palate; he presented with no other neurological deficit such as external ocular movement disturbance, hearing loss, limb paralysis, or ataxia. Screening MR imaging scan including time-of-flight (TOF) MR angiography had revealed infarction in the left cerebellar hemisphere and occlusion of the left vertebral artery (VA), both of which mimicked VA dissection (Fig. 1). His neurological symptoms were deteriorating through conservative treatment, and he was admitted to our department.

He had a history of autoimmune pancreatitis and underwent whole-body ^18^ F-fluorodeoxyglucose positron emission tomography integrated with computed tomography scan, revealing elevated glucose intake at and enlargement of the pancreas, kidney, and bilateral submandibular glands. Serum IgG4 level was markedly elevated (2410 mg/dL). These findings were consistent with IgG4-RD.

### Vessel wall imaging

The patient underwent 3D T1-weighted black blood VWI with and without contrast agent using a 3 tesla MR unit (MAGNETOM Skyra, Siemens Healthcare, Erlangen, Germany) with 32-channel head coil. The imaging parameters were as follows: TR = 1000 ms, TE = 11 ms, field of view = 180 × 180 mm, matrix = 320 × 320, slice thickness = 0.56 mm (0.56 mm isotropic voxel), bandwidth = 380 Hz/pixel, acceleration factor = 2 × 2, and scan time = 5 m 34 s. To improve imaging contrast between the vessel wall and cerebrospinal fluid, the imaging sequence was prepared by DANTE prepulse [13–15]. The parameters of this prototype sequence were as follows: 148 pulse trains, RF pulse flip angle = 10°, and pulse spacing = 1.1 ms with a spoiler gradient applied along the three axes. Three-dimensional gradient echo imaging was performed using the volumetric interpolated breath-hold examination (VIBE) technique to produce another set of 3D anatomical images. Its parameters were TE/TR = 2.29/6 ms, FOV = 230 × 230 mm, matrix = 250 × 250, slice thickness = 0.9 mm (0.9 mm isotropic voxel), receiver bandwidth = 220 Hz/pixel, acceleration factor = 2, and scan time = 2 min 24 s.

**Fig. 2:**
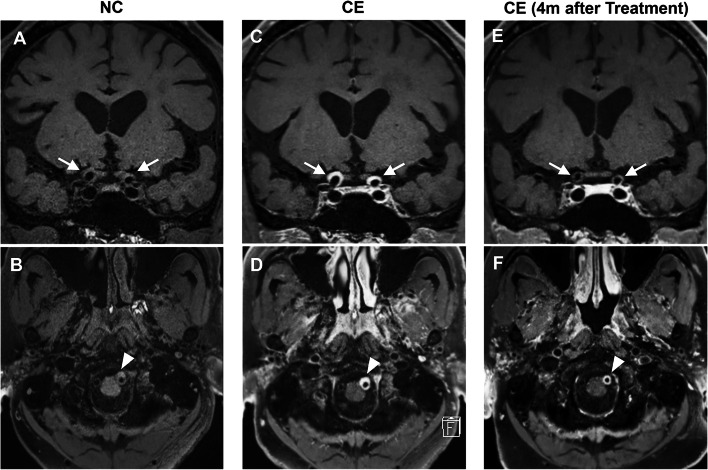
Magnetic resonance vessel wall images (VWIs) with the delay alternating with nutation for tailored excitation (DANTE) prepulse (Case 1). **A** and **B** Non-contrast VWIs on admission. **C** and **D** Contrast-enhanced VWIs on admission. **E** and **F** Contrast-enhanced VWIs acquired 4 months after corticosteroid therapy. Note that nearly-circumferential mural thickening with homogeneous enhancement was observed in supra-clinoid internal carotid arteries (arrows) and left vertebral artery (arrowheads); both lesions were diminished after treatment

VWI revealed lesions not only at the left intracranial VA but also at the supra-clinoid portion of the bilateral internal carotid arteries (ICAs) (Fig. 2). The latter lesion had not been recognized with MR angiography. All these lesions were characterized as nearly-circumferential mural thickening with iso-intensity on non-contrast T1-weighted image, which was homogeneously enhanced with contrast agent (Fig. 2). These findings were incompatible with dissection and were considered to indicate the arteriopathy associated with IgG4-RD. The outer diameter of the ICA was expanded at the supra-clinoid portion, while the luminal space remained intact despite mural thickening (Fig. 2 A and C). A similar expansive remodeling was observed in the left VA at the level below the occlusion (Fig. 2B and D); however, both outer and inner diameters were severely narrowed at the occlusion site.

T1-weighted contrast enhanced MR imaging scan had also revealed enhanced mass lesions at the bilateral lacrimal glands and at the geniculate ganglion of the left facial nerve. The patient’s neurological symptoms were attributable to these lesions and/or direct inflammation spreading from the arteritis to the cranial nerves. Antiplatelet agent was not administered. He did not undergo biopsy of the intracranial vessel wall because the procedure is highly invasive.

He was referred to immunologists, and corticosteroid treatment (prednisolone 40 mg per day) was started. This treatment was tolerated and no adverse event occurred. His symptoms were resolved shortly after the treatment. Serum IgG4 level was significantly reduced (185 mg/dL) 4 months after treatment initiation. VWI performed at the same period revealed decreased mural thickening and enhancement in the bilateral supra-clinoid ICAs (Fig. 2E). A similar improvement was partly observed in the left VA (Fig. 2 F); however, the occlusion itself remained unrestored.

## Case 2

### History

A 65-year-old female complained of pain at the neck, scapula, and left arm. She was treated with analgesia by a general physician; however, the pain gradually worsened, and she was referred to our hospital.

**Fig. 3:**
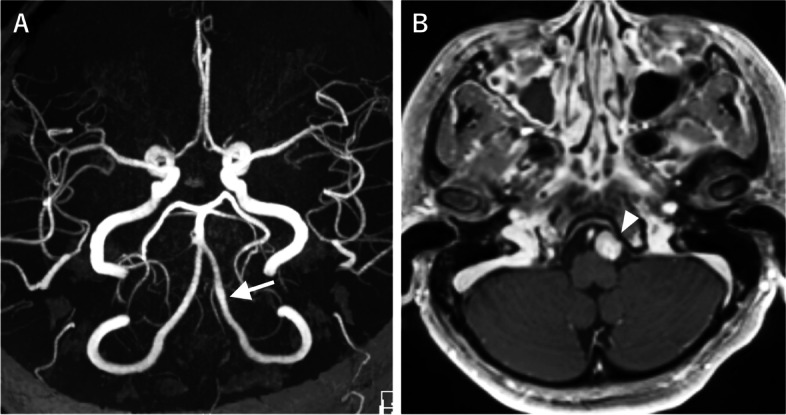
Magnetic resonance (MR) images of Case 2. **A**: Time-of-flight MR angiography revealing only minimal luminal irregularity in the left vertebral artery (arrow). **B**: Contrast-enhanced T1-weighted image revealing a lesion resembling a fusiform aneurysm in the left vertebral artery (arrowhead)

Screening cervical CT scan revealed a tumor mass progressing from the intervertebral foramen to the left brachial plexus. It also incidentally revealed bilateral lacrimal gland enlargement and an unidentified enhanced lesion around the left VA. Serum IgG4 level was elevated (484 mg/dL). TOF MR angiography detected only minimal luminal irregularity at the left VA (Fig. 3 A). On the other hand, contrast-enhanced T1-weighted image revealed an unidentified lesion which resembled a fusiform aneurysm in the left vertebral artery (Fig. 3B).

### Vessel Wall Imaging

**Fig. 4:**
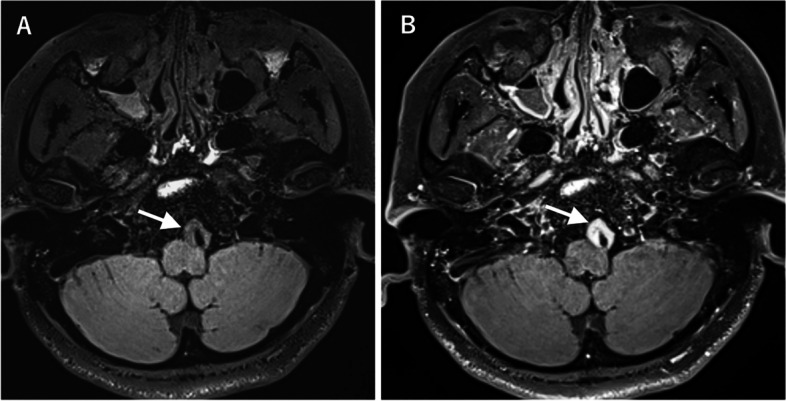
Magnetic resonance vessel wall images (VWIs) with the delay alternating with nutation for tailored excitation (DANTE) prepulse (Case 2). Non-contrast (**A**) and contrast-enhanced (**B**) VWI revealing nearly-circumferential massive mural thickening with homogeneous contrast enhancement at the left vertebral artery (arrows)

DANTE-prepared 3D T1-weighted black blood VWI revealed circumferential massive mural thickening with homogeneous contrast enhancement at the left VA (Fig. 4 A and B), very similar to the findings of Case 1. Lacrimal gland enhancement was also confirmed. A clinical diagnosis of IgG4-RD was made by immunologists. She did not provide informed consent for lacrimal gland biopsy and has been treated conservatively without steroid as her wish. No improvement of the lesion has been observed as of this writing.

## Discussion and conclusions

The present two cases shared a unique finding on VWI: nearly-circumferential mural thickening with homogeneous contrast enhancement. Some lesions had not been recognized with screening TOF MR angiography because expansive remodeling kept the lumen almost intact. The lesions decreased shortly after corticosteroid treatment; this reasonably suggests that there was an association between these lesions and IgG4-RD. As of this writing, this is the first report describing the VWI finding of IgG4-related intracranial arteriopathy.

The present cases are consistent with the literature on IgG4-related intracranial arteriopathy, which exhibits various stenotic and aneurysmal formations. Kondo et al. and Ikeoka et al. reported similar cases, both of which exhibited stenosis of the middle cerebral artery causing infarct [9, 10]. On the other hand, Toyoshima et al. reported a patient who died of vertebrobasilar dolichoectasia possibly associated with IgG4-RD [8]. Marlin et al. also reported a case with diffuse intracranial dilating vasculopathy, which resulted in fatal subarachnoid and intraparenchymal hemorrhage [7]. Interestingly, Nishino et al. reported mural thickening of the bilateral internal carotid arteries resembling one of our cases (*Case 1*) [11]. They focused, however, on dural lesions associated with IgG4-RD and provided less information on the arterial lesions.

The finding of VWI corresponds to the histopathology of IgG4-related arteritis occurring in the coronary artery and aorta. Matsumoto et al. described a case with coronary arteritis, whose histopathology showed circumferential mural thickening with massive infiltration of inflammatory cells including IgG4-positive plasmacytes [16]. They also found that such pathologic change mainly occurred in the adventitia and media. This finding contrasts with atherosclerosis, which predominantly involves the intima. Aortitis associated with IgG4-RD shares the same pathologic feature [17, 18]. Adventitial involvement in IgG4-RD might explain the characteristic expansive remodeling observed in our cases. According to a comprehensive review by Akiyama et al., IgG4-related coronary arteritis is classified into three types: stenotic, aneurysmal, and diffuse wall thickening types [19]. This classification might be applicable to intracranial arteriopathy. Although they do not explain the reason for such variations, we speculate that various proportions of inflammatory cell infiltration between adventitia and media, as well as the original diameter of the vessel, might cause the variations.

VWI with and without contrast agent might facilitate the early detection and differential diagnosis of IgG4-related intracranial arteriopathy, both of which might be challenging with other imaging modalities. Early detection of the pathology is particularly important because it causes fatal sequelae [7, 8] but might be treatable with corticosteroid. Matsusaka et al. reported a case with IgG4-related carotid arteritis detected with ultrasonography, which was improved by corticosteroid therapy [20]. We applied DANTE preparation to VWI; this promising technical modification could enhance the significance of VWI in detecting IgG4-related intracranial arteriopathy through improving imaging contrast [13–15]. The present report has the limitation that histopathology was not confirmed; however, this type of limitation is inevitable in most intracranial pathologies treated medically. Further studies to elucidate characteristic findings of VWI are required to validate the clinical significance of VWI in detecting IgG4-related intracranial arteriopathy.

## Data Availability

The datasets used and/or analyzed during the current study are available from the corresponding author on reasonable request.

## Abbreviations

DANTE: delay alternating with nutation for tailored excitation
ICA: internal carotid artery
IgG4-RD: immunoglobin G4-related disease
MR: magnetic resonance
TOF: time-of-flight
VA: vertebral artery
VWI: vessel wall imaging
